# Exploring Mixed-Reality Exergames for Sports Rehabilitation: Design Insights and Evaluation Findings

**DOI:** 10.2196/68431

**Published:** 2025-10-31

**Authors:** Michelle C Haas, Larissa Wild, Leander Schneeberger, Eveline S Graf, Anna Lisa Martin-Niedecken

**Affiliations:** 1 Institute of Physiotherapy School of Health Sciences ZHAW Zurich University of Applied Sciences Winterthur Switzerland; 2 Institute for Design Research Department Design Zurich University of the Arts Zurich Switzerland; 3 Digital Health Design Living Lab Zurich University of the Arts Zurich Switzerland

**Keywords:** interdisciplinary, exergames, sports rehabilitation, physiotherapy, research and development, user-centered design, evaluation, anterior cruciate ligament

## Abstract

**Background:**

Exergaming, involving physically active play, provides a means for motivating and functional training. It may also offer an innovative solution for rehabilitation after sports injuries such as anterior cruciate ligament (ACL) injury. Rehabilitation is lengthy and only partially prepares athletes for the demands of sports. As a result, only 65% of athletes return to the same performance level as before the injury. Exergames specific to ACL rehabilitation are missing. Their unique combination of physical and cognitive challenge may contribute to preparing patients for their return to sports. Collaboration of sport scientists, game designers, and physiotherapists enables comprehensive and user-centered development of an innovative training concept incorporating the needs of both patients and therapists along with scientific evidence.

**Objective:**

This project aimed to develop a specific exergame scenario for sports rehabilitation after ACL injuries. A research-driven, user-centered, iterative approach was followed. The project was structured into four phases: (1) assessment of motor performance during a fitness exergame, (2) investigation and establishment of user requirements, (3) development of a new exergame, and (4) validation of the new exergame.

**Methods:**

For assessment of motor performance, lower extremity kinematics during a fitness exergame scenario in the ExerCube were assessed in 24 athletes (6 after ACL injury) using marker-based motion capture. Regarding user requirements, focus groups with physiotherapists and patients were conducted. Development of a new exergame was from the kinematic analysis results and user requirements guided the iterative, interdisciplinary design of a new exergame scenario and movement concept for the ExerCube. For validation, developed exergame scenarios were recurrently evaluated with patient and therapist focus groups and transformed into a final exergame scenario.

**Results:**

For assessment of motor performance, there was a main effect of exercise in maximal knee valgus, knee internal rotation, and hip flexion with *P*<.001. Squats showed the lowest knee valgus (4.23°, 95% CI 4.12-4.51) and knee internal rotation angle (3.68°, 95% CI 3.33-4.03). Focus groups revealed that patients want to return to sports as quickly as possible but have concerns about sensory overload during training. Physiotherapists desire a device that allows additional independent training with new therapeutic stimuli. Concerning the development of a new exergame, the movement concept for the new exergame included strength and balance exercises, and a mini-exergame for endurance, reaction, and skill training. Three difficulty levels allow for varying complexity and speed. For validation, focus groups on the newly developed exergame scenarios highlighted their motivational potential, suitability for autonomous use, and the need for therapist-controlled adaptation and integration with conventional tools.

**Conclusions:**

The interdisciplinary, evidence-based approach facilitated a systematic development of requirements for an exergame for rehabilitation, ensuring user-centered implementation. A final evaluation confirmed its motivational potential and applicability, leading to its deployment at all ExerCube locations.

## Introduction

### Rehabilitation After Sports Injuries

Rupture of the anterior cruciate ligament (ACL) is a commonly known sports injury resulting in at least 6 months of time loss until a full return to sport is possible [1-3]. Between 1988-1989 and 2003-2004, the incidence rate (IR) of ACL injuries increased by 1.3% per year among collegiate athletes in the United States [4]. As reporting of sports injuries is not standardized, differences in IRs exist. While Gornitzky et al (2016) [5] report an IR of 0.062 per 1000 athletic exposures among different types of sport, Moses et al (2012) [6] report an IR of 0.05 per average person per year and an IR of 0.15%-3.7% for professional athletes. In female floorball, the risk of an ACL injury equals 3.6 per 1000 game hours [7]. Therefore, injury rates differ depending on factors such as the type or level of sport.

This difference is also present when assessing injury risk for a second ACL rupture, either on the ipsilateral or contralateral side. The latest systematic review and meta-analysis reports a reinjury rate of 21.9% while another meta-analysis reported 15% [8,9]. An even lower reinjury rate, 9.6%, was reported in a prospective study following 1082 patients after ACL reconstruction [10]. The high rates of reinjury suggest that current rehabilitation protocols still have to be improved. Evidence-based rehabilitation protocols are currently mainly available for patients who undergo reconstructive surgery of the ACL. Immediate mobilization, weight-bearing, strength training, and neuromuscular training are recommended after surgery [1-3,11-14]. In contrast, no benefits of bracing could be shown, and it is therefore not recommended [2,11]. Rehabilitation protocols without surgery include proprioceptive training, strength training, and counseling to avoid high-risk movements [12]. To guide progression throughout the rehabilitation process, the concept of “control to chaos” can be followed. This concept provides a structured framework stating that in early stages, exercises with high control of body movement should be performed. As rehabilitation advances, the level of complexity and uncertainty is gradually increased, introducing different stimuli as elements of “chaos” to better reflect real-life scenarios and prepare for a return to sports [15,16].

Only 65% return to their preinjury level of sports after an ACL reconstruction and consecutive rehabilitation [17]. Multiple reasons could explain this low rate: poorly performed surgery or rehabilitation, lax criteria for rehabilitation progression or return to sport, or the lack of addressing psychological factors during rehabilitation [17]. Additionally, patients still exhibit altered movement patterns, which are associated with a higher risk for ACL injury, and diminished explosive neuromuscular performance after ACL rehabilitation [18-21]. According to Buckthorpe (2019) [18], a more holistic and interdisciplinary approach to rehabilitation protocols is needed in order to overcome these deficits. Potentially, exergaming could assist in overcoming these deficits.

### Exergaming

Oh and Yang (2010) [22] define exergaming as “a combination of exertion and video games including strength, balance, and flexibility activities.”

Exergames, which require physical effort and are controlled by (full-body) movements, have been proposed as effective training and therapy alternatives by interdisciplinary research and development communities. These games are designed to be attractive and effective, which makes them suitable for application areas where additional motivation and physical-cognitive training load, as well as a safe environment, are needed [23].

Studies in various target audiences have shown that exergaming has the potential to increase training adherence [24], long-term motivation [25], and engagement [26]. Exergaming has also been found to have positive effects on mental health, including social interaction, self-esteem, self-concept, motivation, and mood [27-32].

Furthermore, exergames can have positive effects on cognitive functions, such as executive functions, attention, and visual-spatial skills [32-37] as well as physical activity levels, energy expenditure, and heart rate. By immersing players in an audiovisual, narrative-based game scenario, exergames shift their cognitive and emotional focus to the playful experience, facilitating engagement in physically challenging training [38,39].

Compared to traditional single-task training or therapy approaches, exergames combine physical and cognitive training and thus provide an incorporated dual task training approach [39], which is beneficial for different target groups [36,40-42].

### Dual Task and Motor Performance

When dual-tasking in a jump landing task by counting backwards, healthy participants (CONs) have shown altered movement patterns such as decreased knee flexion (KF) angles at initial contact, decreased jump height, or increased peak posterior and vertical ground reaction forces in the initial phase of landing [43]. Therefore, landing mechanics associated with increased ACL loading occur when a simultaneous cognitive challenge is added to jump-landings.

In people after musculoskeletal injury, such as an ACL injury, motor performance is also altered when dual-tasking [44]. For gait and postural stability, dual task effects in people after ACL injury have already been investigated and compared to CONs [45,46]. Compared to a single task, patients after ACL reconstruction showed smaller peak KF angles and peak knee extension moments during the loading response phase of walking. Additionally, the difference between legs in peak hip adduction angle (middle stance and terminal stance) and peak hip abduction moment (loading response phase) was decreased in the dual-task condition [45]. Anteropostural stability, as well as overall stability during a standing task, was lower with the additional auditory Stroop task in patients after ACL reconstruction [46]. Overall, compared to CONs, patients after ACL reconstruction sacrifice performance to compensate for the additional cognitive load of a dual task, which could result in increased injury risk.

### Exergaming in Rehabilitation

Extended reality is a continuum that encompasses virtual reality (VR), augmented reality, and mixed reality. While in VR, the user is visually and aurally occluded from the physical environment, in augmented reality, the user interacts with virtual objects in a physical environment. Mixed reality blends the experience of both VR and augmented reality [47]. In rehabilitation, the use of extended reality and serious games is increasing in popularity [48]. In comparison to traditional rehabilitation, using extended reality in rehabilitation provides more excitement as well as physical and cognitive fidelity [48]. Moreover, there is an external focus of attention which leads to a better movement execution [49]. Extended reality devices such as head-mounted displays or monitors can represent immersive systems which, together with physical presence, involvement, and social- or self-presence, lead to an immersive experience [47]. The immersive nature of a digital environment also has positive effects, such as improved movement patterns in patients after ACL injury [50]. Exergames, physically active games performed with extended reality, are known for their promising potential as complementary or alternative rehabilitation tools, but not every exergame is equally suitable. For example, the implementation of an adequate movement concept and cognitive challenges is just as important as the implementation of a motivating and immersive, multisensory game experience. Furthermore, matching the exergame to the needs of the primary (athletes) and secondary (therapists) users is crucial for the successful long-term use of an exergame in sports rehabilitation. While exergames have been used in a variety of clinical settings, such as neurorehabilitation or geriatrics [51,52], their application for sports rehabilitation is very limited [53].

### ExerCube: Physically Immersive Fitness Game Environment

One exergame setting featuring various game and training experiences, which was iteratively designed by involving different target audiences and interdisciplinary research and development teams, is the ExerCube [54-57].

**Figure 1:**
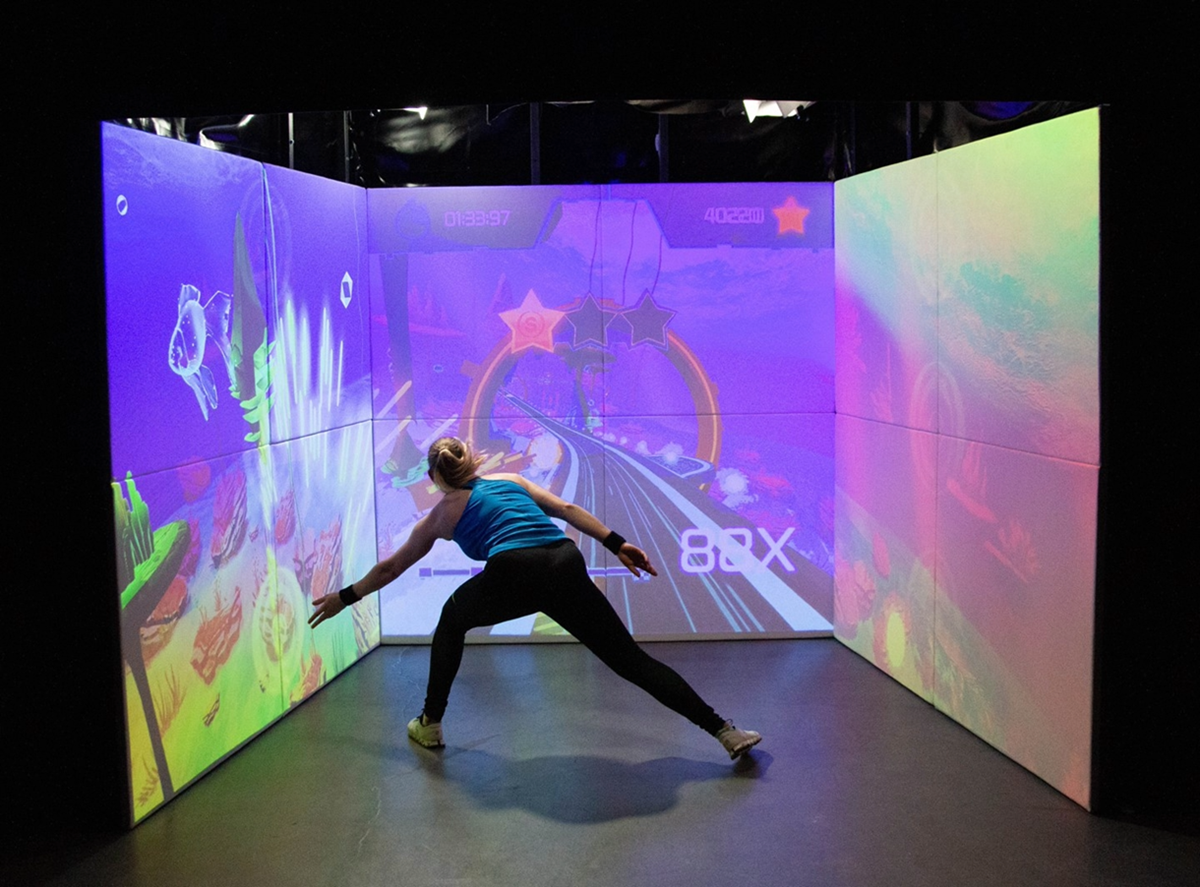
A person playing the exergaming scenario “Sphery Racer” in the ExerCube (source: Sphery Ltd).

The ExerCube consists of a physical immersive training hardware (platform) and several exergame experiences with different physical-cognitive training foci. The Sphery Racer (Figure 1), which was the first exergame experience that could be played on the cube, takes the player on a digital science-fiction underwater race.

The digital game scenario is projected onto the 3 cube walls surrounding the player. To control the game, the player performs functional full-body workout exercises, which are tracked by 4 HTC Vive (HTC Corporation) trackers that the player wears on both wrists and ankles. The game starts with low-to-moderate intensity exercises, which increase to high-intensity exercises over time.

Additionally, the player’s physical (via movement execution) and cognitive performances (via reaction time) are tracked and used to automatically adjust the game’s difficulty and complexity in real time. Generally, the workout intensity in the Sphery Racer is scalable from moderate to high intensity.

Among other things, the Sphery Racer experience was proven to be an attractive and effective alternative to functional high-intensity interval training with a personal trainer [39,58], to provide high exercise intensities (86% of maximum heart rate) [59], and to yield significant positive effects on cognitive (motor) skills (faster reaction times), especially on concentration, cognitive flexibility and divided attention in young game sports athletes after a training intervention [60].

### Goal of This Project

The overarching aim of this project was to design and evaluate a mixed reality exergame specifically for sports rehabilitation. The iterative research and development stages of the interdisciplinary project are presented as follows.

**Figure 2:**
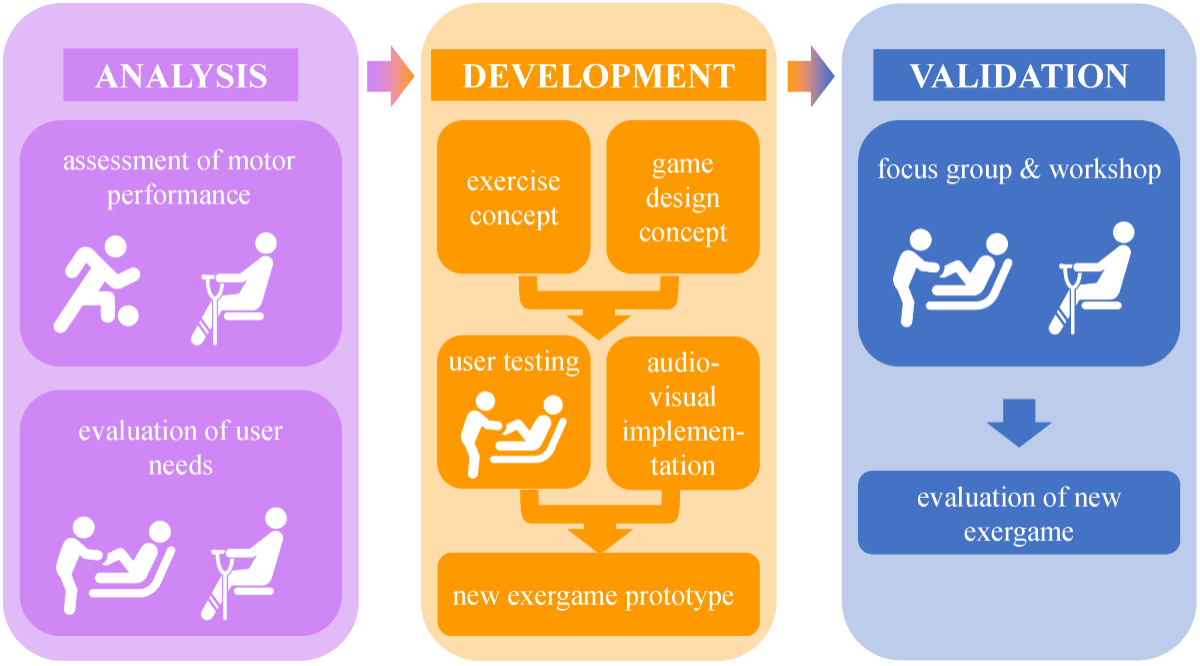
Illustration of the different project phases.

The project consisted of several project phases (Figure 2). As this figure shows, the results of the various phases formed the basis for the subsequent phases. Before the design work could start, motor performance during an existing fitness exergame scenario and user needs for a rehabilitation-specific exergame had to be evaluated. This knowledge was then incorporated into the design work, which included the definition of an exercise concept with the subsequent creation of a game design concept. An iterative cycle of internal testing, feedback from therapists, and implementation of audiovisual cues led to the final prototype of the new exergame. Focus groups and workshops with patients and physiotherapists (primary and secondary end users) were performed to evaluate the prototype. Each section of this paper is divided into subsections that correspond to the 4 different phases.

## Methods

### Motor Performance During ExerCube Training

#### Objective

The goal of this part of the project was to analyze the movement patterns during exergaming to determine during which exercises the participants’ lower extremities are in a more injury-prone position.

#### Participants

Participants were recruited between November 2021 and July 2022 in Winterthur, Switzerland. They were either recruited at the university campus via mail or at local sports clubs via flyers. Before enrolment, participants were screened based on preselected inclusion and exclusion criteria. All participants had to be aged between 18 and 40 years, participate in at least 3 hours per week in a volleyball, handball, soccer, or floorball sports club, and understand verbal and written instructions in German. Exclusion criteria for all participants were an acute or chronic musculoskeletal, neurological, or cardiopulmonary disease, severe pain during exercise execution, and pregnancy. Additionally, CONs was excluded if there was a prior injury to the ACL or prior knee surgery. Patients were only included if they were still in physical therapy but were cleared to fully participate in athletic activities.

In total, 18 CONs (9 females) and 6 patients (2 females) were included. The type of sports was volleyball (n=3), handball (n=5, two patients), soccer (n=9, two patients), and floorball (n=7, two patients). Characteristics of all participants are displayed in Table 1.

**Table 1:** Mean and SD of participant characteristics based on athletes who participated in the biomechanical analysis during exergaming.

Characteristic	Patients (n=6)			CONs^a^ (n=18)			Difference	
	Mean	SD	Mean		SD	Welch *t* test^b^ (*df*)		*P* value
Age (years)	25.00	5.90	25.22		3.26	0.09 (1,6)		.93
Height (m)	1.76	0.07	1.71		0.08	–1.31 (1,10)		.22
Weight (kg)	77.22	2.84	69.07		9.01	–3.37 (1,22)		<.01
BMI (kg*m^–2^)	25.00	1.33	23.42		1.45	–2.47 (1,9)		<.05
Sport-specific training (hours per week)	7.17	3.8	7.58		3.41	0.24 (1,8)		.82

^a^CON: healthy participant.

^b^2-tailed.

#### Data Collection and Analysis

Data was collected in the movement laboratory of Zurich University of Applied Sciences. Both data collection and analysis are described in detail elsewhere [61,62]. Participants performed a 25-minute high-intensity exergame (Sphery Racer, ExerCube) including different exercises. Neutral (side-unspecific) exercises included burpees, jumps, squats, and tripples (skipping). Side-specific exercises were punches, middle touches, high touches, low touches, and lunges. As the game changes speed depending on the participant’s performance, the movement speed and intensity vary depending on the speed of the game. There were 5 levels for speed, and participants needed to perform exercises continuously correctly for 20 seconds to increase the speed level. After each timing or exercise mistake, the participant dropped 1 level. Consequently, the level of speed varied between participants as well as within 1 data collection. The speed of the game was the only variable to change. The order of the exercises was constant for all participants. Critical angles refer to the peak angles of the corresponding joint direction during 10°-30° KF.

#### Statistical Analysis

Data were excluded for the left leg of 1 CONs due to the slipping of marker clusters. Therefore, only data from the right leg was statistically analyzed and will be presented here. First, using data of CONs, it was analyzed during which of the exercises the biomechanical risk factors are more pronounced than in others. Then, data of the 2 exercises most at risk and the exercise least at risk were compared between CONs and patients.

For CONs, a 1-way repeated measures ANOVA with concurrent pairwise *t* test was applied using the package *rstatix* (version 0.7.1) in R (version 4.2.2; R Foundation) [63,64]. For result interpretation, marginal means were estimated and graphically displayed with the package *emmeans* [65]. Maximal angle of either knee valgus (KV), knee internal rotation (KIR), or minimal angle of hip flexion (HF) was the outcome of interest. *Y_i_*, exercise was the within-subject covariate (*exercise_k_*), *β*_0_ represented the intercept, *β_k_* the effect of the k-covariate, and *ε_i_* the independent and normally distributed errors *ε_i_~N*(0,*σ*^2^). The variable exercise had 14 levels, 1 for each combination of side (if applicable) and exercise.

*Y*_i_=*β*_0_+*β*_1_*exercise*+*ε_i_*

No deviations from homoscedasticity or normality were found with residual analysis through visual observation. Significance level was set to *P*<.05.

Comparison of patients and CONs was performed by means of an independent Welch *t* test in the neutral exercise squat and the side-specific exercises, high touch and punch. Significance level was set to *P*<.05.

### User Requirement for Rehabilitative ExerCube Use

#### Objective

The goal of this part of the project was to gain an in-depth understanding of the people directly involved in the rehabilitation after ACL injury (patients and physical therapists), extract their needs and desires during rehabilitation, and make this knowledge accessible to the game designer.

#### Participants

Four physical therapists specialized in sports rehabilitation, as well as 1 athlete who underwent rehabilitation after ACL injury, were purposely chosen to participate in interviews. Additionally, a sports physical therapist was observed during a workday to observe their interaction with various patients with an ACL injury.

#### Data Collection and Analysis

##### Data Collection Methods

Different data were collected to assess user requirements: (1) guideline-based observation in a clinical setting; and (2) guideline-based, semistructured interviews and focus groups.

##### Observation in Clinical Setting

The interaction between patients and physical therapists during ACL rehabilitation was observed by the game designers and movement scientists. The focus of the observation was on the therapeutic content specific to different phases of the rehabilitation and the interaction between the patient and therapist. Subsequently, the observed therapeutic content was matched to phases of a rehabilitative pathway based on functional outcome measures [3,66]. This matching was done by a movement scientist together with the observed physical therapist.

##### Interviews and Focus Groups

Physical therapists (n=4) and 1 patient were interviewed based on a semistructured interview guideline. The physical therapists were interviewed in 2 separate focus groups, while the patient was interviewed alone. The interviews included questions about attitude and experience toward gaming or exergaming (eg, “Have you had experience with exergames?”), therapeutic concepts (eg, “How would you perceive your role as therapist while your patient plays an exergame in the therapy session?”), motivation during rehabilitation (eg, “Which exercises motivate you and feel particularly good, strong, like “achievement” or “powerful”?”), feedback methods (eg, “What kind of feedback (feedback on current performance or over time) do you like in rehab?”), and design preferences (eg, “Would you prefer a realistic or an abstract scenario in the exergame?”).

Three authors with varied disciplinary expertise (human-computer interaction, game research and physiotherapy, and movement sciences) undertook the qualitative evaluation of interviews. Using an iterative thematic coding method inspired by the thematic analysis by Braun and Clark (2021) [67], emerging results were thoroughly discussed until consensus was attained. The results were then transferred into a user persona for both therapists as well as patients who might potentially use the intended prototype.

### Rehabilitation-Specific Motivating Workout Scenarios for the ExerCube

#### Objective

The goal of this part of the project was to develop a movement concept and exergame design concept based on the information from previous project parts to consecutively design a motivating workout scenario specifically for rehabilitation purposes.

#### Iterative Design Process

Following an iterative, interdisciplinary, and holistic design process, a new exergame scenario for the “back to sports” phase of sports rehabilitation after knee injury was developed.

General components of the technological setup of the original setting (cube hardware and tracking with HTC Vive) were kept for the purpose of this project. The movement concept, as well as the software, was iteratively developed. Throughout the iterative concept and prototyping phases, various guided and sequential walkthrough [68] and bodystorming [69] sessions with participants from different backgrounds (physiotherapy, movement science, and game design) were performed. During the sessions, participants were asked to think aloud [70] regarding their experience with the respective concept or prototype. Additionally, participants were observed by the interdisciplinary team and asked questions during the sessions. The team took notes during each session, which were analyzed and discussed afterward from the different disciplinary perspectives and transferred into concrete design decisions.

This iterative procedure enabled the implementation of a new exergame concept consisting of a knee rehabilitation-specific, functional movement concept including motor-cognitive components, as well as a matching, user-centered, engaging audiovisual and narrative game design to be applied in the ExerCube setting, which is described in more detail as follows.

### Validation of the ExerUp Game

#### Objective

The goal of this project part was to obtain feedback as well as first impressions on the newly developed workout scenario by secondary end users.

#### Participants

Physical therapists specialized in sports rehabilitation (N=3) and a personal trainer (N=1) were purposely chosen to participate in a focus group where the newly developed exergame scenario was presented, and their impressions and feedback were recorded.

#### Focus Group

As part of the focus group, all participants were able to (1) observe a person performing a session with the newly developed *ExerUp Game* and (2) try out the game themselves and provide feedback (think aloud) during the try-out, as well as while observing others.

Additionally, a guideline-based semistructured interview was performed focusing on relevant aspects of the digital game scenario, the tracking technology, the movement concept, the overall experience, as well as potential application and implementation cases.

The interviews and the feedback were assessed by 3 authors with different disciplinary backgrounds (human-computer interaction or game research and physiotherapy or movement sciences) following an iterative thematic coding approach based on thematic analysis by Braun and Clark (2021) [67]. Emerging results were discussed until agreement was reached.

### Ethical Considerations

#### Objective

The present study was conducted according to the local legal requirements as well as the guidelines of the Declaration of Helsinki [71] and the principles and procedures for integrity in scientific research involving human beings.

#### Motor Performance During ExerCube Training

This phase of the project was approved by the ethics committee of the canton of Zurich (Basec-No.: 2021-01700). All participants read and signed an informed written consent form before being included in this study. All study data were recorded without identifiable information to ensure privacy and confidentiality. A participant identification list was stored separately from any study data. Participants did not receive any compensation for study participation, though related travel expenses were remunerated.

#### User Requirement for Rehabilitative ExerCube Use, Rehabilitation-Specific Motivating Workout Scenarios for the ExerCube, or Validation of the ExerUp Game

These phases did not require ethical approval as no health-related data were captured. Nevertheless, all study data were recorded without identifiable information (eg, during interviews) to ensure privacy and confidentiality. Participants did not receive any compensation for study participation.

## Results

### Motor Performance During ExerCube Training

#### About CONs

There was a main effect of exercise in all maximal angles of the investigated variables: KV (*F*_13,10958_=133.65, *P*<.001), KIR (*F*_13,10958_=136.50, *P*<.001), and HF (*F*_13,10952_=169.68, *P*<.001).

**Figure 3:**
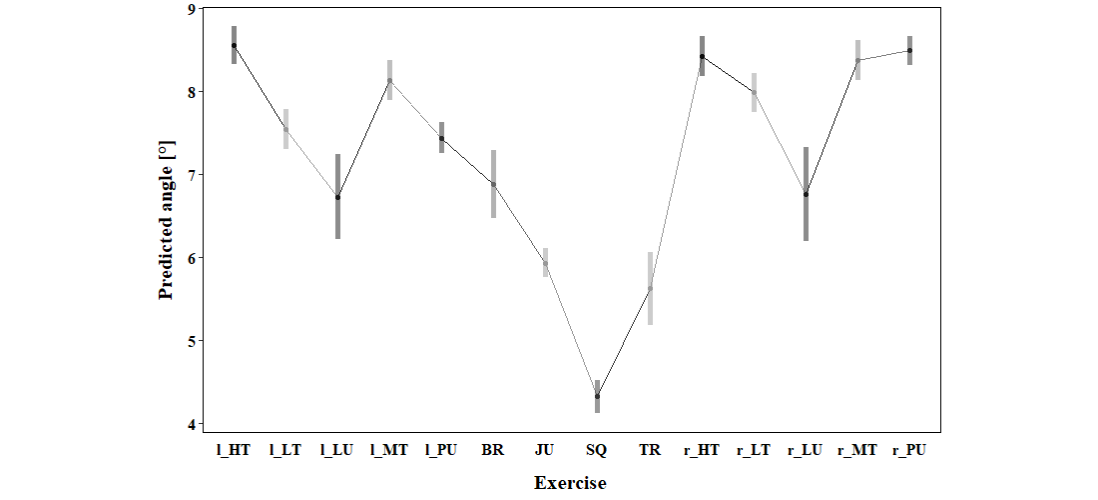
Estimated marginal means for maximal knee valgus of the right leg during 10°-30° knee flexion based on the biomechanical analysis during exergaming of athletes without any prior knee injury. BR: burpee; JU: jump; l_HT: left high touch; l_LT: left low touch; l_LU: left lunge; l_MT: left middle touch; l_PU: left punch; r_HT: right high touch; r_LT: right low touch; r_LU: right lunge; r_MT: right middle touch; r_PU: right punch; SQ: squat; TR: tripples.

**Figure 4:**
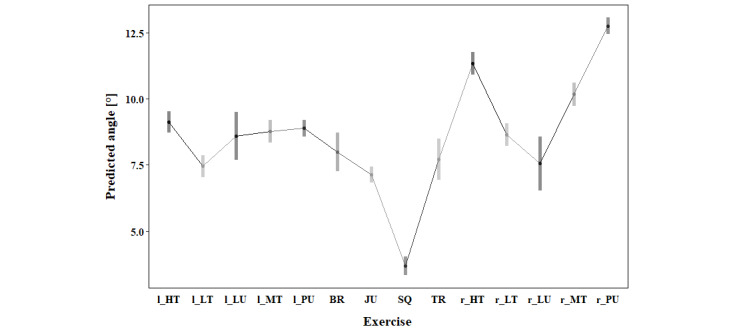
Estimated marginal means for maximal knee internal rotation of the right leg during 10°-30° knee flexion based on the biomechanical analysis during exergaming of athletes without any prior knee injury. BR: burpee; JU: jump; l_HT: left high touch; l_LT: left low touch; l_LU: left lunge; l_MT: left middle touch; l_PU: left punch; r_HT: right high touch; r_LT: right low touch; r_LU: right lunge; r_MT: right middle touch; r_PU: right punch; SQ: squat; TR: tripples.

**Figure 5:**
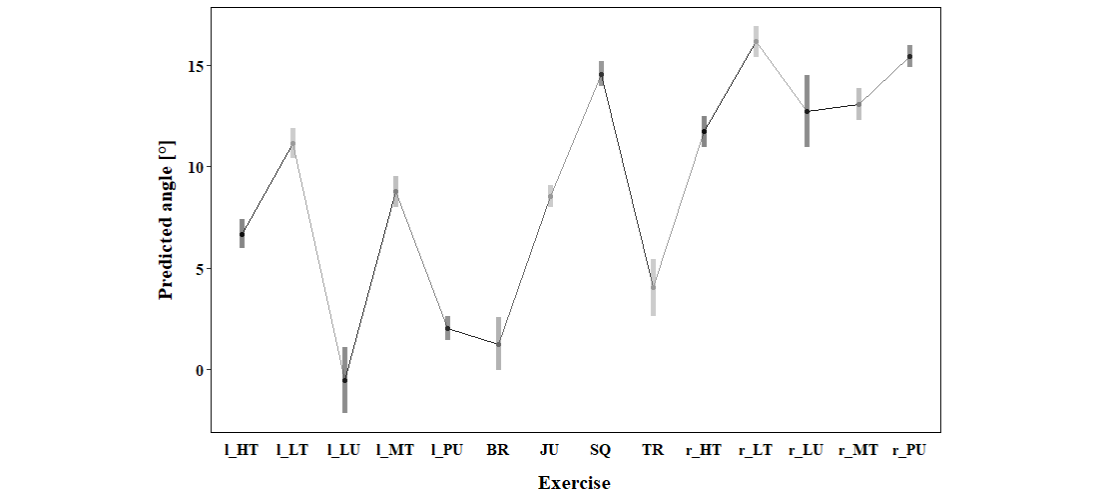
Estimated marginal means for minimal hip flexion of the right side during 10°-30° knee flexion based on the biomechanical analysis during exergaming of athletes without any prior knee injuries. BR: burpee; JU: jump; l_HT: left high touch; l_LT: left low touch; l_LU: left lunge; l_MT: left middle touch; l_PU: left punch; r_HT: right high touch; r_LT: right low touch; r_LU: right lunge; r_MT: right middle touch; r_PU: right punch; SQ: squat; TR: tripples.

Pairwise comparison showed significantly lower KV (*P*<.001) and KIR (*P*<.001) during the squat compared to all other exercises. Moreover, the tripples and jump showed a lower KV (*P*<.001) compared to 11, respectively 9, other exercises. In contrast, the high-touch (both sides), the middle touch (to the right), and the punch (to the right) had a higher KV compared to 8 other exercises (*P*<.001). For maximal KIR, the punch to the right side displayed higher maximal angles than all other exercises (*P*<.001). Additionally, the high touch (to the right) and the middle touch (to the right) displayed higher KIR than 12, respectively 10, other exercises (*P*<.001). A lower HF was present during the left lunge (compared to 11 other exercises), during the left punch and burpee (compared to 10 other exercises), as well as during the tripples (compared to 9 other exercises). A higher HF compared to 11 other exercises was present in the low-touch (to the left), while a higher HF than 10 other exercises was present in the right punch and higher than in 9 other exercises in the squat. An overview of the estimated marginal means, including confidence level for each variable, can be found in Figures 3-5 and in Tables S2-S4 in Multimedia Appendix 1.

#### Patients

In comparison to CONs, patients showed significant differences in maximal KV and KIR during 10°-30° KF of the right leg during all investigated exercises, except for the KV of the right high touch. However, in minimal HF, significant differences between CONs and patients were only found in the squat and left punch (Table 2).

**Table 2:** Comparison of the biomechanical results of different exercises between CONs^a^ and ACL^b^-injured participants.

			CONs (n=18)				Patients (n=6)				Difference		
Location and exercise (side)			Mean		SD	Mean		SD		Welch *t* test^c^ (*df*)			*P* value
**Knee valgus [°]**													
	Squat	4.32		3.48		4.75		3.15	–2.29 (1,745)			.02	
	High touch (left)	8.55		3.54		8.39		2.34	0.86 (1,719)			.39	
	High touch (right)	8.42		3.28		9.38		2.89	–4.53 (1,504)			<.001	
	Punch (left)	7.44		3.71		8.26		3.25	–4.40 (1,808)			<.001	
	Punch (right)	8.49		3.32		9.58		3.29	–6.18 (1,780)			<.001	
**Knee internal rotation [°]**													
	Squat	4.56		6.39		3.21		4.91	6.12 (1,1778)			<.001	
	High touch (left)	6.75		4.59		9.11		5.90	7.00 (1,605)			<.001	
	High touch (right)	11.35		5.78		8.38		5.16	7.85 (1,500)			<.001	
	Punch (left)	8.89		6.80		7.09		5.31	5.68 (1,912)			<.001	
	Punch (right)	12.77		6.96		9.45		7.36	8.51 (1,741)			<.001	
**Hip flexion [°]**													
	Squat	14.32		10.64		16.29		9.54	–4.83 (1,1501)			<.001	
	High touch (left)	6.67		10.43		5.89		9.15	1.20 (1,533)			.23	
	High touch (right)	11.72		10.01		11.87		10.11	–0.22 (1,441)			.83	
	Punch (left)	2.04		11.81		–0.46		10.59	–4.08 (1,758)			<.001	
	Punch (right)	15.42		10.54		14.68		10.02	1.37 (1,797)			.17	

^a^CON: healthy participant.

^b^ACL: anterior cruciate ligament.

^c^2-tailed.

#### User Requirement for Rehabilitative ExerCube Use Evaluation Outcomes

The observation in the clinical setting, as well as the focus groups, revealed that there is a need for a motivating enhancement of conventional physiotherapy. Conventional physiotherapy can provide effective interventions, but it is challenging to keep patients motivated over the long-term period of rehabilitation after an ACL injury. This is especially true after about 6 months of rehabilitation, when progress may slow down and more complex tasks need to be trained, for example, with agility drills, jumping and hopping exercises, and full strength and balance need to be obtained. In this phase, it would be important to introduce sport-specific, motor-cognitive tasks; however, it is often not possible to allocate sufficient time for such exercises during the limited time for patient-therapist interaction. Further, as patients have different levels of activity preinjury and very different goals for rehabilitation, a digital intervention needs to be adjustable for different levels of performance.

The specific requirements for an exergaming solution for ACL rehabilitation based on patient and therapists’ feedback are that the patient should be independent in its use, while the therapist should be able to manage and monitor the activity. Both physical and cognitive tasks should be challenging with increasing complexity. The exergame should provide feedback to the patient and provide a form of social interaction, which could be competitive or cooperative.

**Figure 6:**
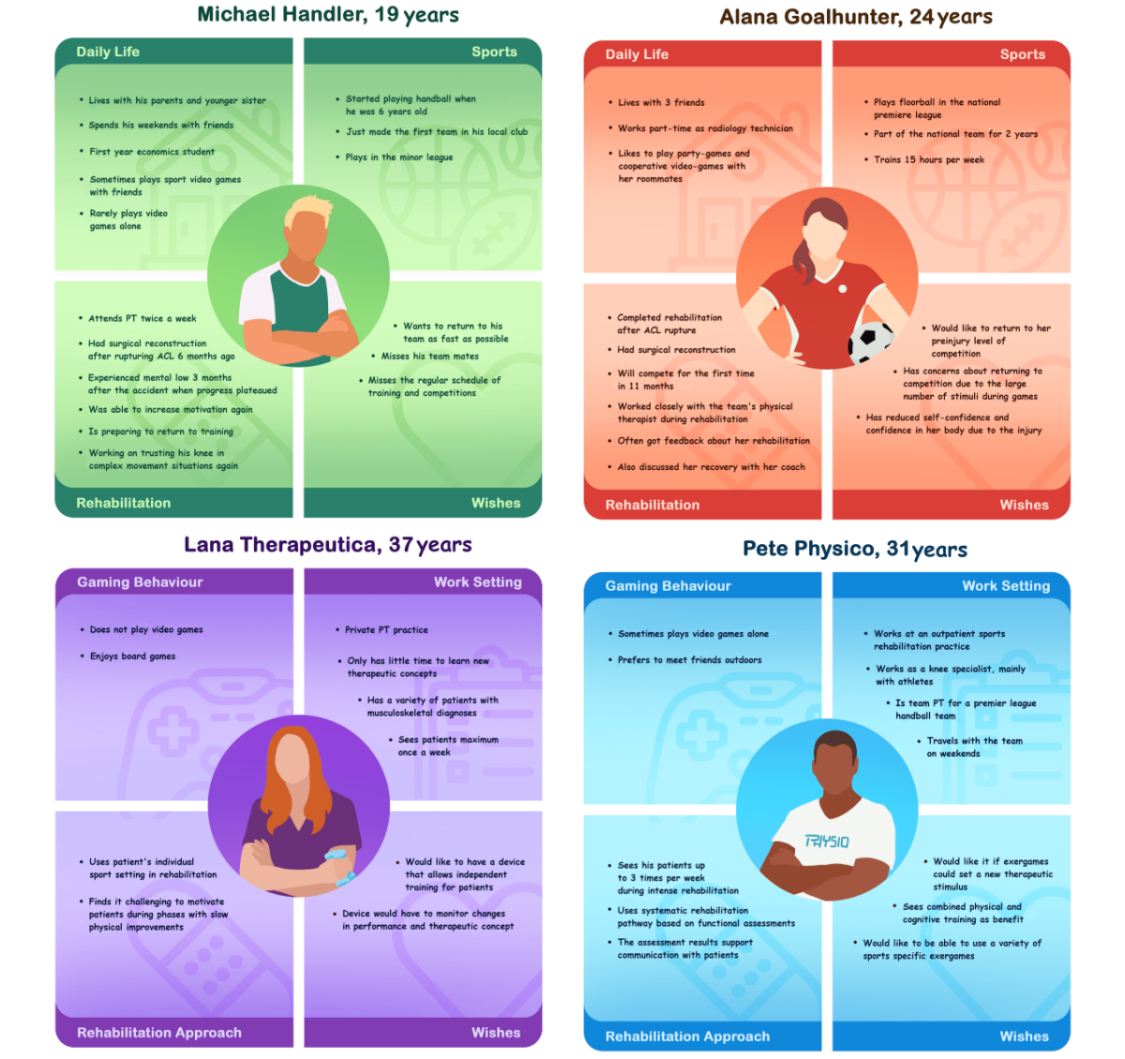
Personas for athletes or patients undergoing rehabilitation and physical therapists were developed based on observations, interviews, and walkthroughs with patients and therapists. ACL: anterior cruciate ligament; PT: physiotherapy.

Based on the results of the observations, interviews, and walkthroughs, personas for patients as well as therapists were developed (Figure 6). Two athlete personas and 2 personas describing therapists were defined, representing a spectrum of individual personal characteristics but also rehabilitation paths and expectations.

### Rehabilitation-Specific Motivating Workout Scenarios for the ExerCube

#### Movement Concept

Based on the findings from the biomechanical analysis of the Sphery Racer [72-77], different exercises were excluded as they are not suitable for sports rehabilitation after knee injury, whereas the findings from the focus groups with patients (primary users) and physios (secondary users) as well as the consultation of experts from the field of movement science and physiotherapy allowed us to derive and connect different functional exercises which are well known for being used in traditional sports rehabilitation after knee injury, and which were then combined in a sports rehabilitation-specific functional movement concept featuring the following foci and movements:

Focus 1 included (1) balance and stability, which used side steps and touches, single-leg deadlift, skater jumps, and lunges; and (2) strength and reaction, which used tuck jumps, burpees, and lateral hops.

Focus 2 included coordination, agility, reaction or speed, and motor-cognitive performance, which used free, dynamic movements.

#### Exergame Experience Design

Furthermore, the focus groups revealed target group-specific preferences and requirements for the look and feel, for motivational aspects, as well as for meaningful game mechanics and gameplay.

**Figure 7:**
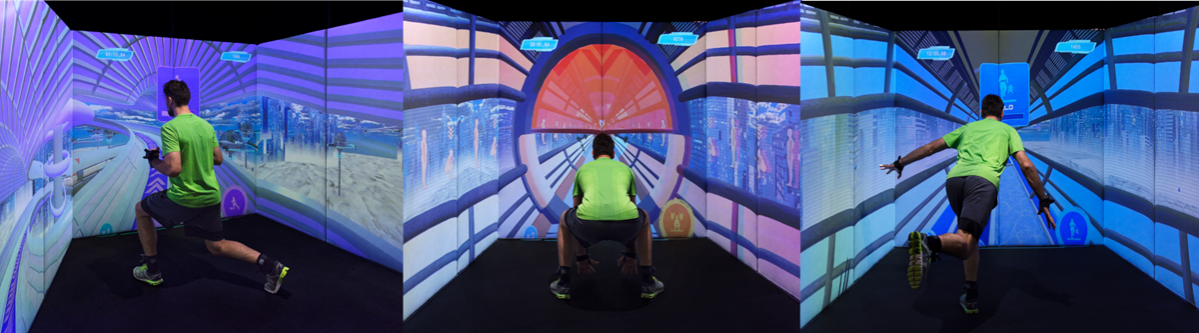
The Tube-Game of the newly developed exergame scenario being played in the ExerCube (source: ZHAW Zurich University of Applied Sciences).

**Figure 8:**
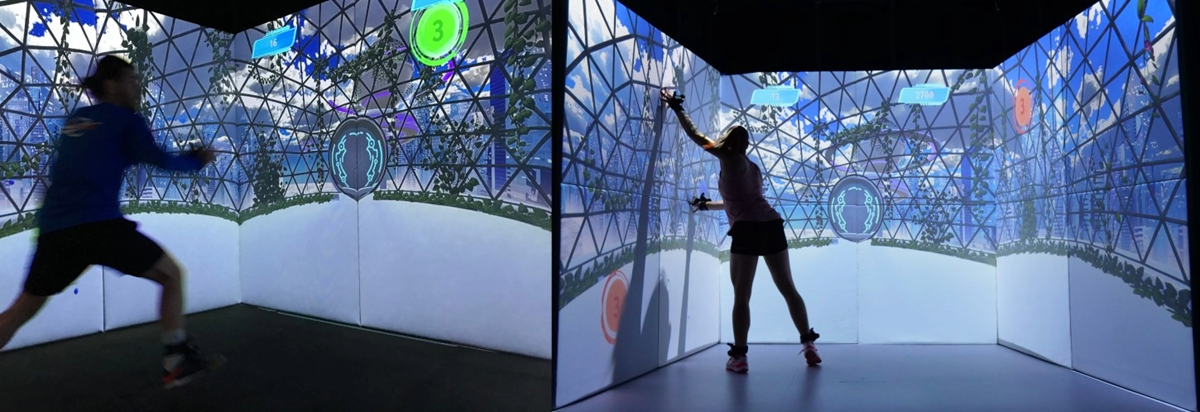
The Dome-Game of the newly developed exergame scenario being played in the ExerCube (source: Zurich University of the Arts and ZHAW Zurich University of Applied Sciences).

Therefore, two complementary exergame concepts were derived: (1) concept 1: clean and sterile environment + little number of stimuli (Figure 7), and (2) concept 2: natural rather overloaded environment + many stimuli (Figure 8).

#### Exergame Description

Following the movement concept as well as the implications for the gameplay, game mechanics, and the audiovisual exergame design were translated into 2 meaningful exergame experiences with slightly different foci. Both experiences provide athletes with an immersive stimulus that allows them to shift their often very internal focus on the body part that was previously injured and set and keep it in their sports environment, such as with their team members, opponents, or a sports device. This is crucial for athletes to be ready to perform safely and well when returning to their sports.

The new exergame scenario (ExerUp-Game) consists of different parts—a main game (Tube-Game, Figure 7) and a mini game (Dome-Game, Figure 8). Both exergame parts focus on different training aspects:

The Tube-Game (control) mainly focuses on jumps, balance, stability, and coordination, whereas the Dome-Game (chaos) focuses on agility, reaction, cognition, and inhibition. Depending on the respective focus of the therapy, the games can either be used sequentially and in combination or in a stand-alone version.

The ExerUp-Game features different presets of intensity and complexity (easy, medium, or hard), which allow patients and therapists to individually choose the matching intensity.

To match the transition from a secure and controlled environment in sports rehabilitation to the specific physical, cognitive, and mental requirements on the field (eg, during a match), the ExerUp-Game represents the principle of “control to chaos” [15].

When playing the ExerUp-Game, the player is elevated onto a platform in a nature-inspired science-fiction world. The player will then be performing the differently colored and challenging exercises to control the avatar on the digitalracing track (Tube-Game). After a while, the player will then enter the Dome-Game*,* which takes the player into a digital sphere, providing differently colored digital targets (green, yellow, and red) on all 3 cube walls surrounding the player, which need to be reached in a short time (includes a countdown). Red targets need to be avoided.

The game plays in a nonsports-specific scenario because it should be suitable for different athletes from different sports disciplines. A video snippet of the ExerUp-Game is available in Multimedia Appendix 2.

### Validation of the ExerUp-Game

#### Objective

The participant’s feedback was organized into different categories, depending on the design component or topic it was related to.

#### Attitudes Toward Gaming and Exergames

In addition to the feedback directly related to design and implementation, participants shared more general attitudes toward gaming and exergames. Some linked the use of games to nostalgic memories from their childhood, suggesting a personal familiarity that could enhance engagement. At the same time, others expressed that they do not intuitively associate gaming with physiotherapy, highlighting a perceptual disconnect between playful digital environments and structured therapeutic contexts. Nevertheless, the use of exergames was seen as a potentially motivating intervention. Particularly for individuals who are typically less engaged in conventional physical activity.

#### Game Content or Training Concept

The 2 parts of the game, Tube-Game and Dome-Game, were rated as being suitable for 2 different time points during rehabilitation, with the Tube-Game being appropriate earlier during rehabilitation than the Dome-Game. Additionally, the Dome-Game was deemed to be complementary to conventional physical therapy as it allows for a training regimen that is not possible in the conventional setting.

Further, it was highlighted that the games are not sport-specific. While this was considered a nonissue, as the games may be complementary to sport-specific training, it remains unclear whether the potential learnings from the games can be transferred to the sport-specific scenarios that the athletes will face after the rehabilitation.

Additional comments indicated that the focus of patients during gameplay could shift away from movement quality and toward game success, raising questions about whether the games sufficiently support therapeutic movement goals. Participants also questioned whether and how the system’s technological structure influences movement execution, and how this aligns with physiotherapeutic best practices.

#### Implementation in Rehabilitation

The therapeutic and training experts deem the games to be playable by the patients themselves, which would free timely, cognitive, and physical resources of the therapists or trainers. The games could be played before or after conventional therapy. For the games to be supplementary to the conventional therapy, they should be adjustable by the therapists so they can define which exercises and which intensity a patient needs to train at. Further, the therapists should receive a report outlining which exercises are performed badly or well in order for the therapists to track the progress of an individual patient. However, the participants reported that they do not see a benefit in integrating the game into the conventional therapy session.

Additionally, participants expressed uncertainty regarding the role of the therapist within the exergame context, in particular, how much influence they could exert on the gameplay and exercise selection, and how visible or trackable therapeutic outcomes would be for them. These ambiguities point to a need for clearer therapist interfaces and configurable training modules that ensure transparency and flexibility in exercise design.

#### Use of Traditional Therapeutic Devices

Overall, the participants see potential to include traditional, therapeutic devices such as balls, weights, or agility ladders in the exergame and expressed the benefit if examples of such combinations of devices are provided by the manufacturer. The simplicity of the use of the ExerCube, together with the potential of individualizing the setting, for example, in combination with traditional devices, was mentioned as a strong benefit of the exergame.

This point was further emphasized through calls for more guidance and best-practice examples from the manufacturer. The integration of analog equipment was seen as a promising way to bridge the gap between familiar physical therapy routines and the novel experience of digital, game-based movement training.

## Discussion

### Principal Findings

The aim of this study was the development of an exergame for knee rehabilitation in sports based on the needs of users, such as patients and therapists, as well as biomechanical principles. Given the motivating environment provided by exergames, there is tremendous potential to disrupt the lengthy and repetitive rehabilitation process that can lead to low motivation [78] and consequently improved adherence [48], which is crucial for good rehabilitation outcomes [79,80]. Additionally, exergames allow for motor-cognitive training that has positive effects on executive functions [60], which are impaired in patients after ACL injury [81].

### Motor Performance During ExerCube Training

This project part aimed to analyze which of the exercises performed during exergaming in CONs elicit the most pronounced biomechanical risk factors associated with ACL injury. Moreover, data of the 2 exercises most at risk and the exercise least at risk were compared between CONs and patients.

Movement strategies and, therefore, maximal KV, KIR, and minimal HF differ between the 14 different exercises. Squat, jump, and tripples demonstrated the lowest peak angles of biomechanical risk factors for ACL injury. In contrast, punch, middle touch, and high touch showed higher KV and KIR angles than the other exercises, indicating a greater potential for ACL strain. Therefore, these exercises present more pronounced biomechanical risk factors for ACL injury and should be performed at a later stage of rehabilitation. If several different levels of the new exergame are to be designed, punches and touches should only be incorporated in the level intended after strength and neuromuscular control are sufficiently restored. Patients in rehabilitation after an ACL injury move differently than CONs during the exercises squat, punch, and high-touch. Patients present with more KV and less HF than CONs. In contrast, patients showed less KIR, suggesting a complex interplay of compensatory strategies. Overall, in 2 of the 3 investigated biomechanical risk factors for an ACL injury, patients showed more extreme movement patterns associated with injury risk. Of the 6 patients, only 2 were female, which could possibly explain the differences to CONs. An analysis of sex differences using data of CONs showed that females move with more KIR than males during touches and punches [62,75]. However, differences between males and females were dependent on the exercise and side, which supports the main effect of exercise in this analysis. Hence, the difference might result from the imbalance of the sexes in the patient group, whereas the CONs group had the same number of male and female participants. Additionally, high interindividual variability in movement patterns, as indicated by the high SDs, could have contributed to the difference [61]. As the analysis was limited to the right leg, a bias related to leg dominance might have been introduced. Incorporating biomechanical analysis into exergame design can help ensure that patients are challenged appropriately at each stage of recovery.

### User Requirement for Rehabilitative ExerCube Use

This qualitative evaluation aimed to develop an understanding of the people directly involved in the rehabilitation after ACL injury, namely the athletes and the physical therapist. Further, key aspects of the rehabilitation after ACL injury were defined to inform the game designers.

In the early phases of rehabilitation, controlled movements are being performed. With the progression of the rehabilitation, more complex and chaotic tasks can be performed with the focus shifting from stability to stop-and-go and 3D, powerful movements as suggested by literature [15,82]. To ensure that an exergame is appropriate for the different levels of performance between athletes, as well as for the changing capabilities of a specific athlete, the game should include different levels of physical and cognitive challenge. A physical therapist should be able to choose which level of challenge to assign to a specific patient at a specific time point during the rehabilitation. Remaining with the concept of “control to chaos,” both more controlled movements with limited cognitive challenge, as well as complex or chaotic movement patterns in combination with high cognitive demand, should be possible with the exergame. In a further step, adaptive difficulty of the motor and cognitive demand would be beneficial to specifically tailor the game to the needs of an individual athlete.

Maintaining motivation and, therefore, adherence to exercising appears to be one of the key challenges during the rehabilitation process after ACL injury [79]. With exergames being highly motivating [48], there could be tremendous potential, especially when the exergame targets a rehabilitation phase where motivation is generally low. The results indicate that elements such as feedback, as well as interaction with other athletes, for example, through leaderboards, could be ways to maintain the motivation of the athletes.

### Rehabilitation-Specific Motivating Workout Scenarios for the ExerCube

The framework guiding the development of the exergaming scenario was chosen as “control to chaos,” which aligns with the suggested progression from controlled movements while building strength and neuromuscular control to more complex and reactive movements later in rehabilitation [82]. Focusing on balance and stability incorporated with the exercises side steps and touches, single leg deadlift, skater jumps, and lunges is especially important at earlier stages of rehabilitation, while later on the focus shifts to strength and reaction, which is incorporated by the exercises tuck jumps, burpees, and lateral hops. Incorporation of the focus on agility, reactivity, and motor-cognitive performance as a second focus corresponds well to the needs of patients and therapists. To create a motivational environment, the look and feel was derived from the requirements of patients and therapists while incorporating an immersive stimulus, shifting the focus externally. Literature suggests that an external focus of attention is beneficial to elicit safer movement patterns when jumping [49]. Application of the workout scenario was intended to be broad; hence, no sport-specific scenarios were incorporated, thus limiting the transfer to sport-specific tasks.

### Validation of the ExerUp-Game

Generally, the newly developed ExerUp-Game was well received. Participants were questioning whether exergames could be a beneficial alternative or addition to traditional physical therapy. The following three aspects were mentioned as crucial: (1) does the ExerUp-Game allow physical therapists to modify the content, (2) does the exergame technology ensure adequate movement quality and safety, and (3) do patients have an internal or external focus of attention (eg, focus on the game or their movement execution) during the exergame session as this determines the time point of the application of the ExerUp-Game or the individual Dome-Game or Tube-Game during the rehabilitation.

### Overall

Combining the detailed analysis of the kinematics during exergaming with user requirements established the need for different levels of physical and cognitive challenge during an exergame for sports rehabilitation. Different movement tasks performed during the exergame were associated with different knee angles. The assessment of qualitative user-specific needs indicated the need for different levels of challenge, starting from controlled situations and ranging to chaotic movement patterns. This information was translated into a movement concept and a design concept that were the baseline for the development of the new, target group-specific ExerUp-Game consisting of 2 individual, but combinable games (Dome-Game and Tube-Game).

To subsequently implement the exergame into the daily practice of the target group, re-evaluation of whether target-specific requirements were met is needed. Only based on the results of an evaluation can the exergame be further improved and find its application in practice.

### Limitations

Each phase of the project had specific limitations, which are outlined below in the order in which the phases are presented in this paper. First, data on biomechanical movement patterns were collected in a realistic setting. Therefore, exercises and their execution and performance were neither normalized nor standardized. As a result, high variability of biomechanical variables was observed. Second, due to the adaptive speed levels of the Sphery Racer according to exercise performance, participants performed a different number of exercises. However, the exercise sequence was fixed, which is why comparability between exercises is still feasible. Third, the peak angle within 10°-30° KF does not necessarily correspond to the peak angle during the entire trial. According to literature, the injury mechanism of ACL ruptures is likely multiplanar [83], justifying the assessment of multiple risk factors simultaneously. Fourth, the sample size was small and unbalanced between CONs and patients, which limits the generalizability of the results of this first project part. Future studies should aim for larger and more balanced samples to strengthen representativeness.

Fifth, in the second part of the project, there were only a few physical therapists and only 1 athlete after an ACL injury was involved. The reason for this was to find experienced people with availability to participate in this project phase. Consequently, individual opinions may have largely influenced the results of interviews and focus groups.

Sixth, there is a lack of scientifically valid and universally accepted rehabilitation pathways after ACL injury. Consequently, the work in the third project phase, the development of rehabilitation-specific workout scenarios for the ExerCube, could not be based on a standardized rehabilitation program. This limits the evidence base used for this work.

Seventh, with the evaluation, it was not established how effective the new rehabilitation game is, what its long-term benefits are, and how it could be implemented in sports rehabilitation. While initial feedback from focus groups was positive, these statements have limited generalizability. These aspects will be covered in future research.

### Recommendations

The development of an exergame for sports rehabilitation requires an interdisciplinary team. As team members often come from diverse professional backgrounds, this can lead to specific challenges. For example, establishing a common terminology often demands extensive discussions and frequent exchanges to avoid misunderstandings. Allocating sufficient resources to address these challenges should therefore be an integral part of planning and implementing similar projects in the future.

Additionally, a user‑centered design approach is essential. A thorough understanding of the needs of both primary and secondary end users is crucial to creating a meaningful exergaming experience that is truly tailored to the target population.

### Conclusions

An interdisciplinary, evidence-based approach combining quantitative and qualitative methods led to the development of a tailored exergame scenario for sports rehabilitation following ACL injuries. Subsequent evaluations with end users indicated that the scenario has strong potential to be both motivating and suitable for independent use. However, further adaptations under the supervision of therapists and closer integration with conventional rehabilitation tools are still necessary.

## Appendix

Multimedia Appendix 1 Detailed information on the performed exercises and biomechanical outcomes.

Multimedia Appendix 2 Video snippet of the newly developed exergame for sports rehabilitation.

## Abbreviations

ACL: anterior cruciate ligament
CON: healthy participant
HF: hip flexion angle
IR: incidence rate
KF: knee flexion
KIR: knee internal rotation angle
KV: knee valgus angle
VR: virtual reality

## Data Availability

Data used for biomechanical analysis is available on the web [84].
