# Supplementary material for: Exploring Mixed-Reality Exergames for Sports Rehabilitation: Design Insights and Evaluation Findings
Source: JMIR Serious Games. 2025 Oct 31;13:e68431. doi: 10.2196/68431 (PMC12619019; doi:10.2196/68431)
Supplement: Multimedia Appendix 1 [file games_v13i1e68431_app1.docx]

Anhang full story paper

**Table S1.**

| Exercise Name | Description of Movement | Illustration from Exergame |
| --- | --- | --- |
|  |  |  |
| Lunge | - Aiming at the corner with the respective foot and holding the arms in place with the hands in front of the chest | 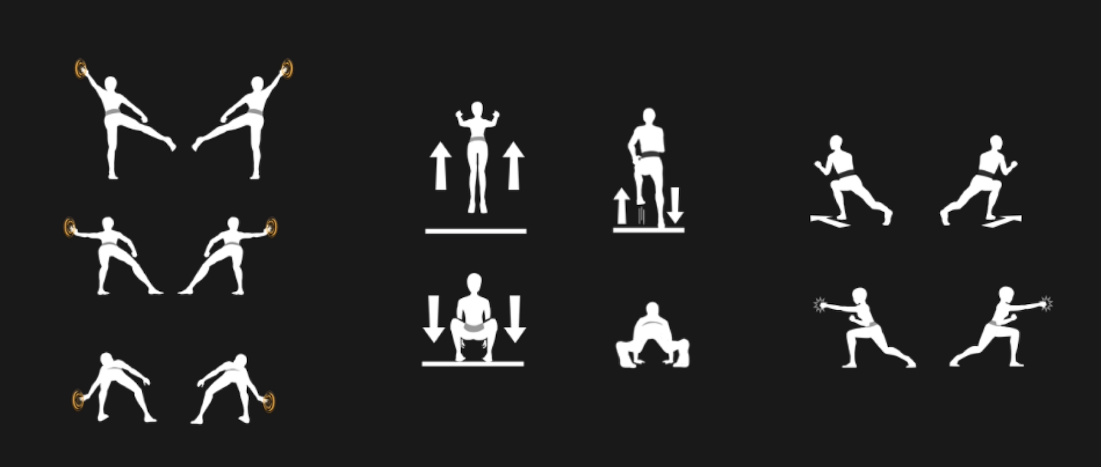 |
| Punch | - Sidestep to the right or left wall - Rotation of the upper body - Punching the wall at shoulder height - Sidestep back to the center | 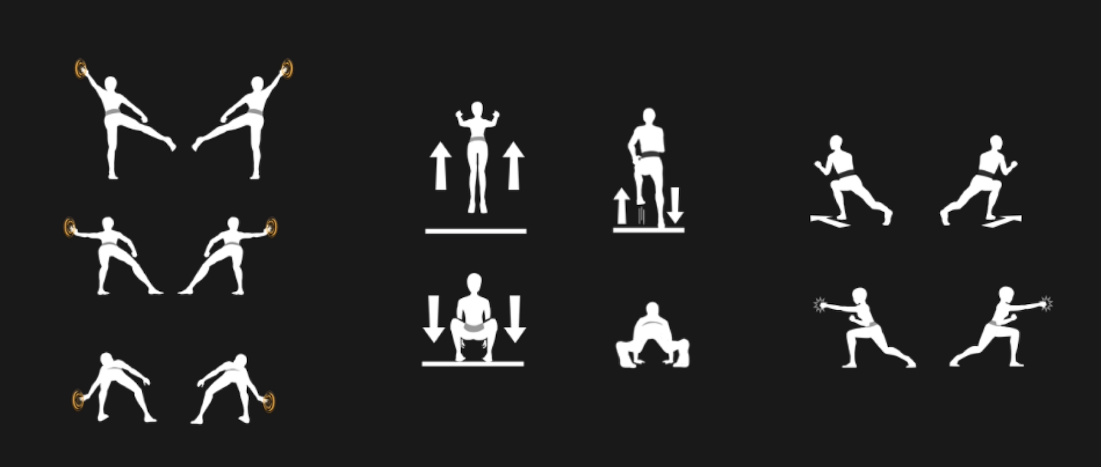 |
| Low Touch | - Sidestep to the right or left wall - Touching the right or left wall near the floor - Sidestep back to the center | 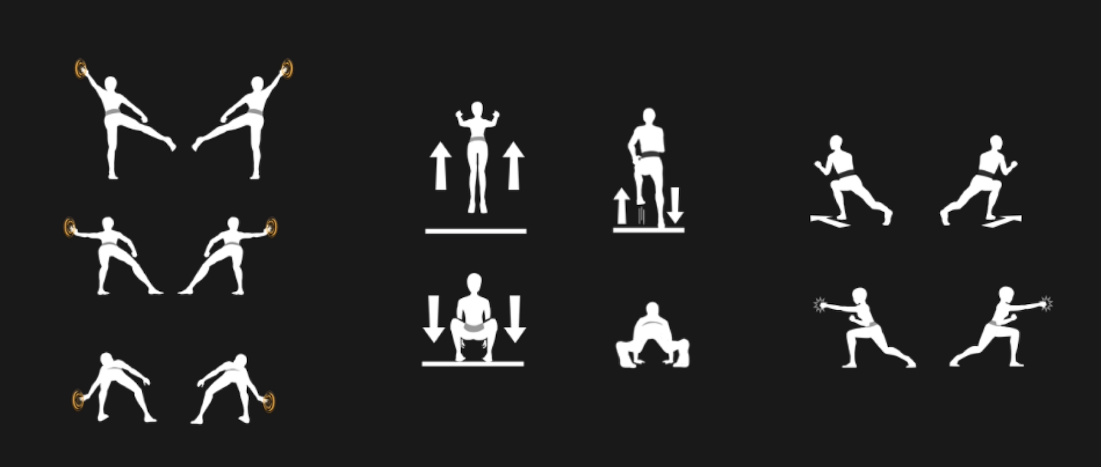 |
| Mid Touch | - Sidestep to the right or left wall - Touching the right or left wall at shoulder height - Sidestep back to the center | 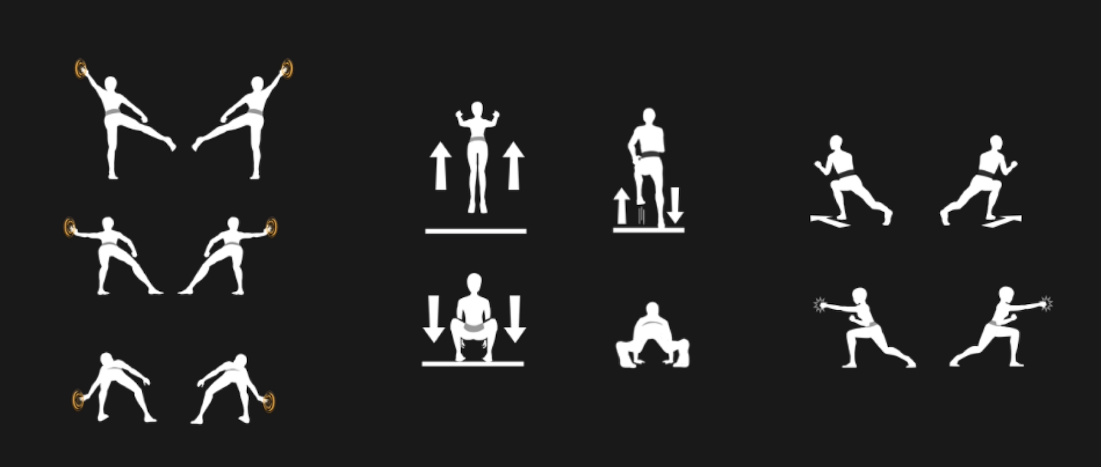 |
| High Touch | - Sidestep to the right or left wall - Touching the right or left wall above shoulder height - Sidestep back to the center | 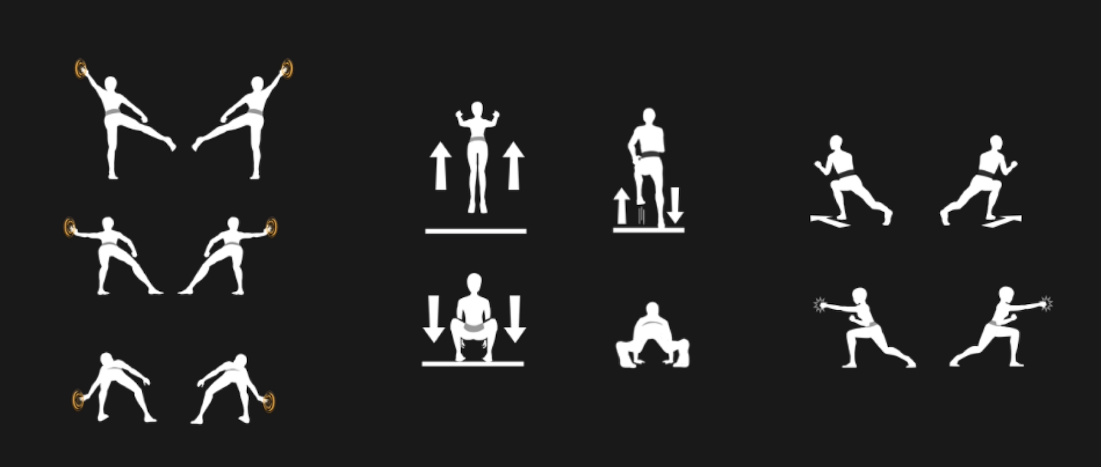 |
| Jump | - Jump in the center of the ExerCube space with arms reaching into the air as high as possible | 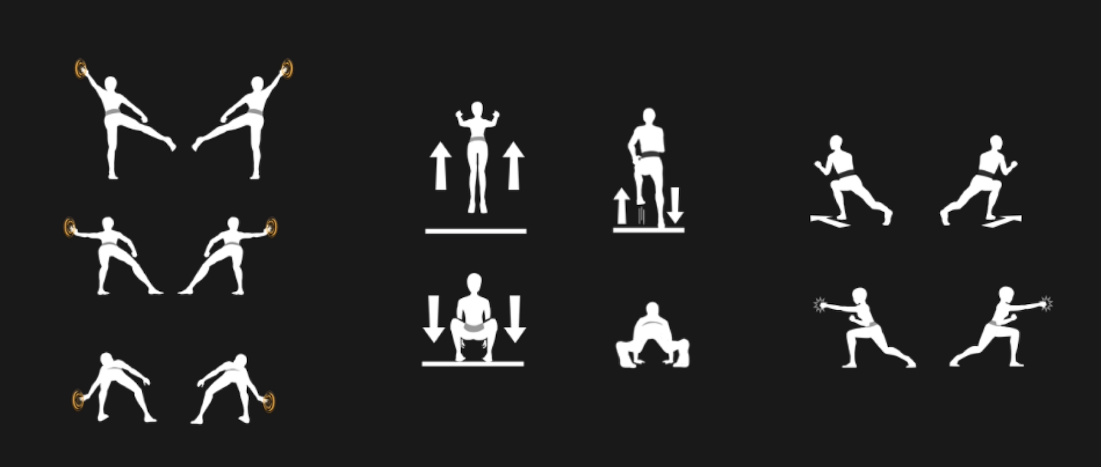 |
| Burpee | - Jump with both hands reaching into the air - Moving in the plank position - Holding the plank position until the speaker says “get up now” | 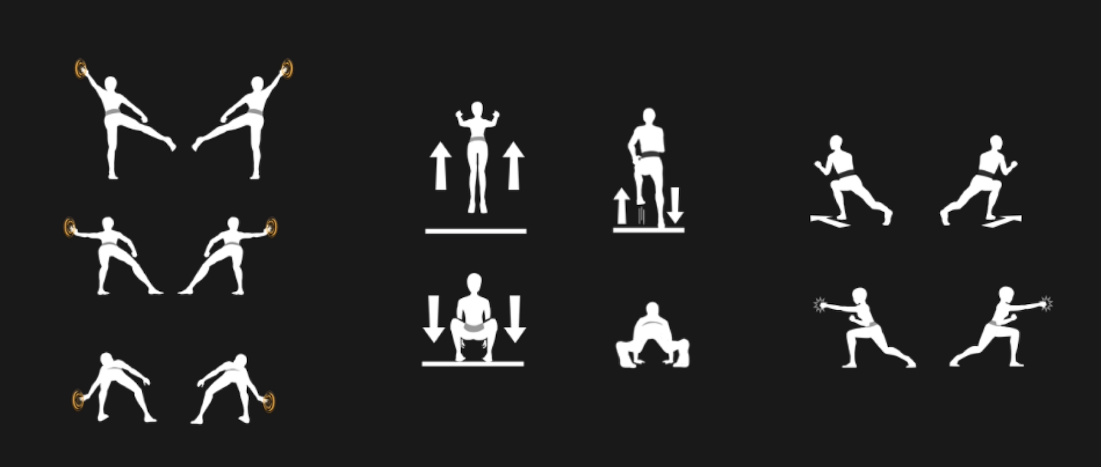 |
| Squat | - Sumo squat in the center of the ExerCube with hands touching the ground - The upper body is kept straight | 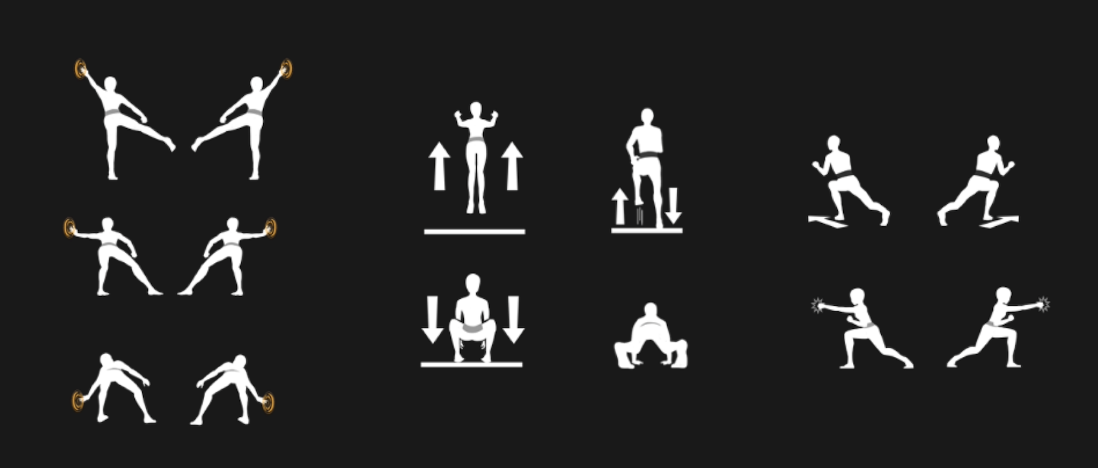 |
| Tripples | - Skipping on the spot in the center of the ExerCube | 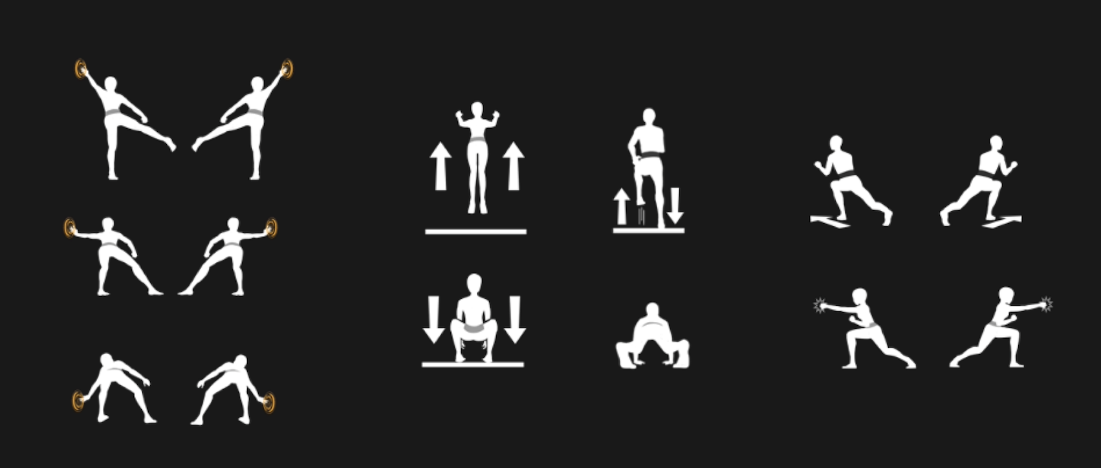 |

**Table S2.** Estimated means maximal knee valgus during 10-30° knee flexion – right leg.

| Exercise | Estimated mean [°] | Standard error | Lower confidence level [°] | Upper confidence level [°] |
| --- | --- | --- | --- | --- |
| High Touch Left | 8.55 | 0.12 | 8.33 | 8.78 |
| Low Touch Left | 7.54 | 0.12 | 7.30 | 7.78 |
| Lunge left | 6.73 | 0.26 | 6.21 | 7.24 |
| Mid Touch Left | 8.13 | 0.12 | 7.89 | 8.37 |
| Punch Left | 7.44 | 0.09 | 7.25 | 7.62 |
| Burpee | 6.87 | 0.11 | 6.47 | 7.28 |
| Jump | 5.93 | 0.09 | 5.76 | 6.10 |
| Squat | 4.23 | 0.10 | 4.12 | 4.51 |
| Tripples | 5.62 | 0.23 | 5.18 | 6.06 |
| High Touch Right | 8.42 | 0.12 | 8.18 | 8.65 |
| Low Touch Right | 7.98 | 0.12 | 7.74 | 8.22 |
| Lunge Right | 6.75 | 0.29 | 6.19 | 7.32 |
| Mid Touch Right | 8.37 | 0.13 | 8.12 | 8.61 |
| Punch Right | 8.49 | 0.09 | 8.31 | 8.66 |

**Table S3.** Estimated means maximal knee internal rotation during 10-30° knee flexion – right leg.

| Exercise | Estimated mean [°] | Standard error | Lower confidence level [°] | Upper confidence level [°] |
| --- | --- | --- | --- | --- |
| High Touch Left | 9.11 | 0.21 | 8.71 | 9.52 |
| Low Touch Left | 7.45 | 0.22 | 7.02 | 7.88 |
| Lunge left | 8.60 | 0.47 | 7.68 | 9.51 |
| Mid Touch Left | 8.78 | 0.22 | 8.35 | 9.21 |
| Punch Left | 8.89 | 0.17 | 8.56 | 9.22 |
| Burpee | 7.99 | 0.37 | 7.26 | 8.72 |
| Jump | 7.14 | 0.16 | 6.83 | 7.45 |
| Squat | 3.68 | 0.18 | 3.33 | 4.03 |
| Tripples | 7.72 | 0.40 | 6.93 | 8.51 |
| High Touch Right | 11.35 | 0.22 | 10.93 | 11.78 |
| Low Touch Right | 8.65 | 0.22 | 8.22 | 9.07 |
| Lunge Right | 7.55 | 0.52 | 6.54 | 8.56 |
| Mid Touch Right | 10.18 | 0.22 | 9.74 | 10.62 |
| Punch Right | 12.77 | 0.16 | 12.45 | 13.09 |

**Table S4.** Estimated means minimal hip flexion during 10-30° knee flexion – right leg.

| Exercise | Estimated mean [°] | Standard error | Lower confidence level [°] | Upper confidence level [°] |
| --- | --- | --- | --- | --- |
| High Touch Left | 6.67 | 0.36 | 5.96 | 7.38 |
| Low Touch Left | 11.14 | 0.38 | 10.39 | 11.89 |
| Lunge left | -0.51 | 0.82 | -2.13 | 1.10 |
| Mid Touch Left | 8.78 | 0.39 | 8.02 | 9.53 |
| Punch Left | 2.04 | 0.29 | 1.46 | 2.61 |
| Burpee | 1.27 | 0.66 | -0.12 | 2.56 |
| Jump | 8.52 | 0.28 | 7.97 | 9.07 |
| Squat | 14.55 | 0.31 | 13.93 | 15.16 |
| Tripples | 4.04 | 0.72 | 2.64 | 5.43 |
| High Touch Right | 11.72 | 0.38 | 10.97 | 12.47 |
| Low Touch Right | 16.14 | 0.38 | 15.39 | 16.90 |
| Lunge Right | 12.71 | 0.91 | 10.92 | 14.49 |
| Mid Touch Right | 13.06 | 0.39 | 12.28 | 13.83 |
| Punch Right | 15.42 | 0.28 | 14.87 | 15.98 |
